# A Device-Free Indoor Localization Method Using CSI with Wi-Fi Signals

**DOI:** 10.3390/s19143233

**Published:** 2019-07-23

**Authors:** Xiaochao Dang, Xuhao Tang, Zhanjun Hao, Yang Liu

**Affiliations:** 1 College of Computer Science and Engineering, Northwest Normal University, Lanzhou 730070, China; 2 Gansu Internet of Things Engineering Research Center, Lanzhou 730070, China

**Keywords:** indoor positioning, channel state information, device-free, wireless, wavelet domain denoising

## Abstract

Amid the ever-accelerated development of wireless communication technology, we have become increasingly demanding for location-based service; thus, passive indoor positioning has gained widespread attention. Channel State Information (CSI), as it can provide more detailed and fine-grained information, has been followed by researchers. Existing indoor positioning methods, however, are vulnerable to the environment and thus fail to fully reflect all the position features, due to limited accuracy of the fingerprint. As a solution, a CSI-based passive indoor positioning method was proposed, Wavelet Domain Denoising (WDD) was adopted to deal with the collected CSI amplitude, and the CSI phase information was unwound and transformed linearly in the offline phase. The post-processed amplitude and phase were taken as fingerprint data to build a fingerprint database, correlating with reference point position information. Results of experimental data analyzed under two different environments show that the present method boasts lower positioning error and higher stability than similar methods and can offer decimeter-level positioning accuracy.

## 1. Introduction

With the continuous development and popularization of wireless communication technology, Wi-Fi has become more and more widely used in daily life, and there is also a growing demand for the location-based service (LBS) [1]. In the outdoor environment, some mature satellite positioning systems, such as Beidou Satellite Navigation System and GPS, will provide users with more accurate location services, and also meet the positioning requirements of most outdoor environments [2]. However, in indoor environments, due to weak satellite signal, poor penetration, and other reasons, the satellite positioning system cannot work effectively. For example, the satellite positioning system can locate mobile devices to a specific building, but not to the floor or room. Therefore, indoor positioning came into being. Due to the limited and complex indoor space and the high accuracy and stability of positioning requirements, a series of wireless signal-based positioning methods, such as Wi-Fi [3], Bluetooth [4], and Ultra-Wide Bandwidth (UWB) [5], have been widely studied and used.

The Wi-Fi-based indoor positioning system has great advantages. In recent years, due to the development of visible light, visible light positioning is also used in indoor positioning [6]. If a complete set of facilities for positioning is built indoors, some high-cost equipment (hardware used for sensing, anchor nodes installed in fixed positions indoors, etc.) will be used, which is not conducive to the popularity of indoor positioning and wastes a lot of resources. Therefore, technologies such as Bluetooth and UWB are not suitable for indoor positioning systems with high precision, high reliability, and low cost. In recent years, Wi-Fi has been widely used in indoor positioning systems due to its low cost, wide signal transmission range, and strong applicability. A typical Wi-Fi-based indoor positioning system usually includes a transmitter and receiver. The transmitter can be a wireless router, and the receiver can be a Wi-Fi-enabled device [7,8]. Under such conditions, there is no need to deploy specific expensive equipment, and the Wi-Fi-based indoor positioning system has a greater advantage than other indoor positioning methods [9].

According to different acquisition signals, Wi-Fi-based indoor positioning technology can be divided into indoor positioning technology based on RSSI signal and indoor positioning technology based on Channel State Information (CSI) [10,11]. However, in the indoor environment, the Received Signal Strength Indication (RSSI) signal, as a kind of coarse-grained information, is highly susceptible to interference from other signals and the indoor multipath effect, so it cannot provide sufficient accuracy and reliability [12,13,14,15]. For Wi-Fi signals using IEEE 802.11n communications protocol, it can obtain CSI in Orthogonal Frequency Division Multiplexing (OFDM) subcarriers by modifying the wireless network card driver. CSI resolved from the physical layer can describe the channel characteristics and state between the signal transmitter and the receiver [16,17]. The discovery of CSI led to the study of indoor positioning technology. It portrayed the multipath transmission to a certain extent, and added the amplitude and phase information of each subcarrier. Compared with RSSI for indoor positioning, CSI provides more fine-grained signal characteristic information, which improves the accuracy of indoor positioning to some extent [18,19,20].

Passive sensing is an emerging technology that senses changes in the environment without the need for any additional equipment [21,22,23,24]. Of course, if human behavior is perceived, the perceived person does not need to wear any equipment. At present, passive sensing has a wide range of research fields, including indoor positioning [25], human behavior perception [26], and intrusion detection [27]. For indoor positioning, the passive sensing application makes the indoor positioning application scenarios broader, such as car-locating in the underground parking lot, navigation of related departments in the hospital, smart home, and so on [28,29,30,31].

According to the principle of positioning, Wi-Fi-based indoor positioning technology can be divided into positioning based on the transmission model and fingerprint-based positioning. Positioning based on the transmission model requires the establishment of an accurate channel model to estimate the distance between the target and each AP (access point). However, due to the large difference between the physical model and the actual transmission environment of signals, and the existence of some interference factors that cannot be estimated by the physical model, the positioning effect is often not as high as that of the fingerprint-based positioning method. The fingerprint-based positioning method is generally divided into an offline phase and an online phase. In the offline phase, the real state information of reference points in the real environment is collected, and the fingerprint database is built after data processing. In the online stage, the status information of test points is collected in real time, and the positioning result is finally obtained by comparing the data processed with the data in the fingerprint database. Since a large amount of state information in the real environment is collected, the fingerprint location method can reduce the impact of environmental changes on the positioning results to some extent, resulting in a higher accuracy of positioning results.

FIFS [32] leverages the CSI values, including different amplitudes and phases at multiple propagation paths, and uses the weighted average CSI values to improve the performance of the RSS-based method. In DeepFi [33], a deep learning-based indoor fingerprinting localization system is proposed. In the offline stage, it chooses to use deep learning to train the collected CSI. In the online stage, a probabilistic method based on the Radial Basis Function is employed to determine the location of the target. The authors of reference [34] designed the novel dynamic multiple signal classification (Dynamic-MUSIC) method to detect the subtle reflection signals to identify the human target’s angle. In PhaseFi [35], a phase fingerprint identification system for indoor positioning, a deep network was designed with three hidden layers to train the calibrated phase data. In BiLoc [36], the authors developed a deep learning-based algorithm to exploit bimodal data. Both the estimated angle of arriving (AOA) and average amplitudes of two adjacent antennas are used as location features for building the fingerprint database.

In conclusion, this paper proposes a passive indoor positioning system based on CSI. The system first collects the CSI through the hardware device, and then preprocesses the collected data. The preprocessing is divided into the amplitude processing and phase processing of the CSI. The amplitude is denoised by a wavelet, the phase is unwrapped and corrected, and the phase is linearly changed. In the offline phase, after the feature information of each reference point is extracted, the amplitude and phase are simultaneously stored as fingerprint information in the fingerprint database. In the offline phase, the real-time data collected are preprocessed and compared with the data in the fingerprint database to obtain the final positioning results.

The contributions of this paper are summarized as follows:

1. In order to ensure the quality of fingerprint data and improve the positioning accuracy, this paper carried out preliminary experiments in the early stage. By setting the environmental state under three different conditions, the method proposed in this paper was verified. Two different environments are set to compare the method proposed in this paper with other methods. The method proposed in this paper is relatively stable in the environment with a strong multipath effect and ordinary environment, which verifies the robustness of the method proposed in this paper to some extent;
2. The effects of the number of packets, the number of reference points, and the data quality on the experimental performance are analyzed separately. The performance of the proposed method under different conditions is considered comprehensively. Finally, the optimal state is selected, which improves the positioning accuracy to some extent and reduces the whole complexity of the method implementation;
3. In the data collection stage, we choose the data in the best quality link among multiple links as the original data, which eliminates the data greatly affected by the environment to some extent and improves the data quality. As far as we know, this is the first time that a filtering of raw data has been proposed;
4. In order to verify the effectiveness of the proposed method, several mature methods at home and abroad are compared from the aspects of actual location effect, location accuracy, and error analysis. Experiments show that the proposed method is superior to the existing methods.

The rest of the paper is organized as follows: Section 2 describes the preliminary. Section 3 describes in detail how to design the system. Section 4 introduces the experimental environment and analyzes the performance of this method through experiments and compares it with other mature indoor location methods. Meanwhile, error analysis is added to analyze the influence of different factors on location accuracy from different stages, thus demonstrating the effectiveness of the proposed method. Finally, we conclude the work in Section 5.

## 2. Preliminary

### 2.1. Channel State Information

For Wi-Fi signals that use IEEE 802.11n communications protocol, the OFDM modulation is used. For signals transmitted by OFDM, CSI analyzed from the physical layer can be used to describe the channel characteristics and states between the transmitter and receiver of signals. OFDM is a method to encode digital data onto several carriers of different frequencies, and has been widely used in the field of wireless communication [37,38,39,40,41,42,43]. It divides the channel into several orthogonal subchannels, converts a high-speed data stream to a low-speed parallel data stream at the transmitter, and modulates the corresponding subchannels for transmission. At the receiver, the received signal is demodulated and restored to the original data. In OFDM transmission system, the frequency domain model of a channel state can be expressed as:

(1) $$\vec{Y}=H\vec{X}+\vec{N},$$

where $\vec{Y}$ and $\vec{X}$ represent the receiving and sending signal vectors respectively, H represents the channel information matrix, and $\vec{N}$ represents the additive white Gaussian noise. The CSI of each subcarrier can be expressed as:

(2) $$H=\frac{\vec{Y}}{\vec{X}},$$

where H varies and the size of the matrix varies according to the hardware. In the hardware equipment using the Inter 5300 network card, if the number of subcarriers is 30, H can be expressed as $H=[H_{1},H_{2},\cdots ,H_{30}]$. For hardware equipment using Atheros 9380 series network cards, if the number of subcarriers operating at 20 MHz is 56, H can be expressed as $H=[H_{1},H_{2},\cdots ,H_{56}]$, and if the number of sub-carriers operating at 40 MHz is 114, H can be expressed as $H=[H_{1},H_{2},\cdots ,H_{114}]$. The CSI of a single subcarrier can be expressed as:

(3) $$H=\left|H\right|e^{j\sin(∠H)},$$

where |H| and ∠H represent the amplitude and phase of corresponding subcarriers, respectively.

**Figure 1:**
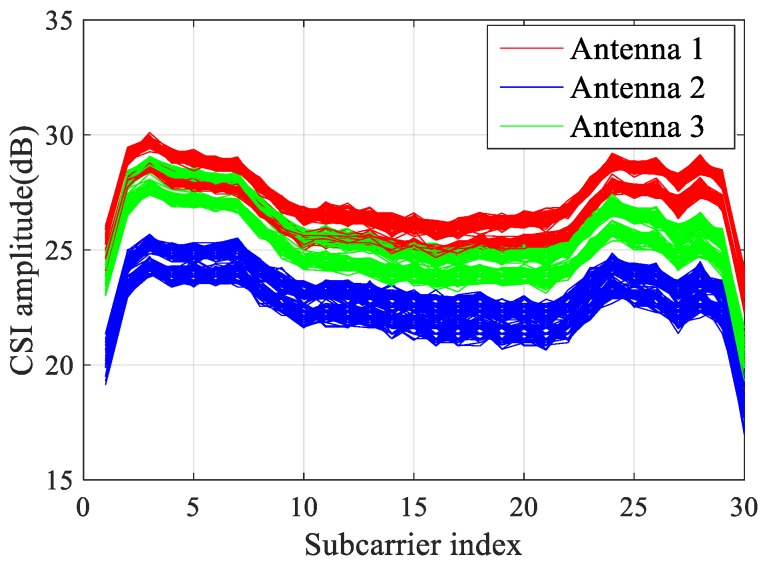
Channel State Information (CSI) Signals.

CSI data comes from a complex matrix m×n×i, in which m and n respectively represent the number of transmitting antennas and receiving antennas, and i represents the number of subcarriers. For each CSI data set, it is composed of a real part and an imaginary part, and also depicts the amplitude and phase of a subcarrier. In this paper, the hardware equipment of the Inter 5300 network card is selected as the experimental equipment, so the number of subcarriers collected from CSI data is 30, as shown in Figure 1.

### 2.2. Data Selection

**Figure 2:**
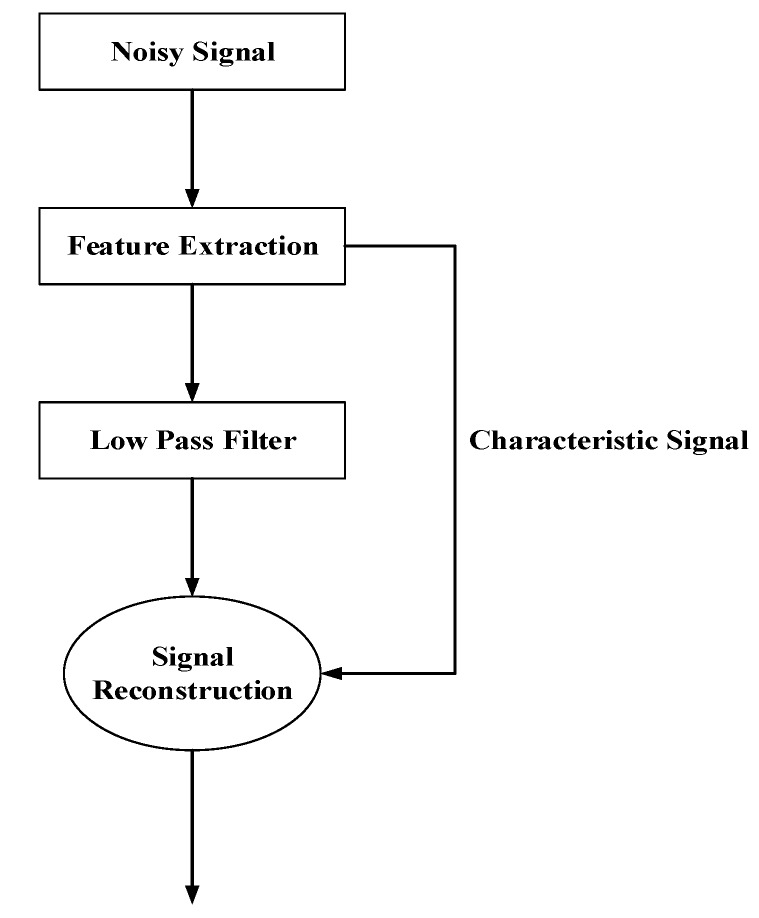
Flow Chart of Wavelet Domain Denoising (WDD).

After the CSI data are collected, the data are preprocessed. CSI data are divided into amplitude and phase. Therefore, the amplitude and phase should be processed separately during preprocessing. First, the amplitude processing is performed, and the trend of the amplitude can indicate different positions. However, since the amount of collected data is large, the influence of environmental noise is large, so denoising is performed. An ordinary filter can remove some noises, but it will cause incomplete signal features. Therefore, in this paper, Wavelet Domain Denoising (WDD) is adopted to deal with the collected CSI amplitude in order to solve the above problems [44,45]. After the signal is transformed by the wavelet, the wavelet coefficients generated by the signal contain important information. After the signal is decomposed by the wavelet, the wavelet coefficient is larger and the wavelet coefficient of noise is smaller, and the wavelet coefficient of noise is smaller than that of signal. By selecting an appropriate threshold, the wavelet coefficients greater than the threshold are considered to generate the signals and should be retained, while those less than the threshold are considered to generate noise and should be set to zero to achieve the purpose of denoising. Although wavelet domain denoising can be regarded as lowpass filtering to a large extent, it is superior to the traditional lowpass filter because it can successfully retain signal characteristics after denoising. It can be seen that wavelet domain denoising is actually the integration of feature extraction and lowpass filtering, and its flow chart is shown in Figure 2. After the input of the noisy signal, the signal is processed by lowpass filtering again in the feature extraction stage, and then the processed signal and characteristic signal are reconstructed to get the final result. A model with noise can be expressed as:

(4) S(k)=f(k)+ε·e(k),

where S(k) is the signal with noise, f(k) is the useful signal, e(k) is the noise, and ε is the standard deviation of the noise coefficient. CSI amplitude information collected from reference points is stored in the fingerprint database after the wavelet domain denoising.

Furthermore, the choice of phase is crucial. CSI data collected from reference points contain phase information, but the original phase information cannot describe the corresponding position information well and needs to be processed. In this paper, the original phase information is corrected by unwinding and then being linearly changed to obtain the phase that can better reflect the position information of the reference point. For an explanation of specific processing methods, please refer to Section 3.2.2.

After the above processing, the amplitude after wavelet domain denoising and the phase after linear change are selected as fingerprint data in this paper, which can better depict the information of each reference position and improve the positioning accuracy.

## 3. System Design

### 3.1. System Overview

**Figure 3:**
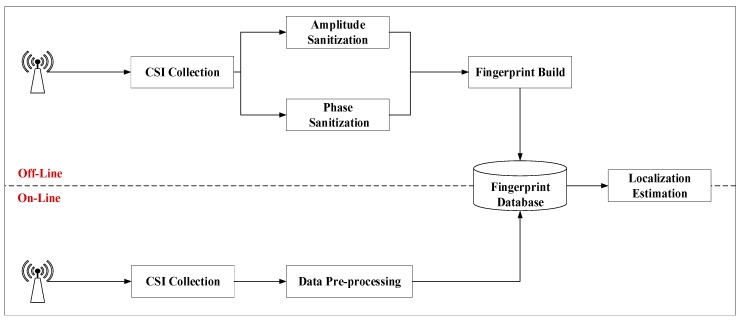
System Framework.

As shown in Figure 3, the whole system is divided into two parts: Offline stage and online stage. In the offline phase, CSI data collection is firstly carried out, followed by data preprocessing. In the preprocessing phase, it is further divided into the amplitude preprocessing phase and phase preprocessing phase. The feature classification is then stored in the fingerprint database. In the online stage, CSI data collection should be carried out first, and the collected data should be preprocessed in the same way as in the offline stage, and the processed data should be compared with the data in the fingerprint database to finally obtain the positioning result. In order to reduce the interference caused by changes in indoor environment to CSI signals, we tried to ensure that the testers were stationary and the test environment was stable. When collecting data in the offline phase to build the fingerprint database and when conducting positioning matching in the online phase, the surrounding environment remained consistent and the furniture position remained unchanged.

### 3.2. Data Pre-Processing

#### 3.2.1. Amplitude Sanitization

**Figure 4:**
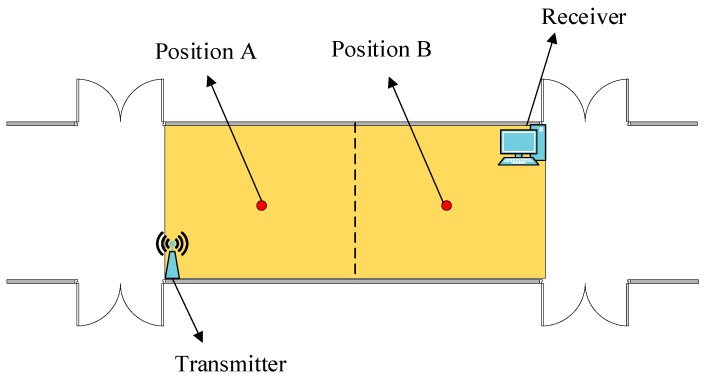
Testing Environment.

**Figure 5:**
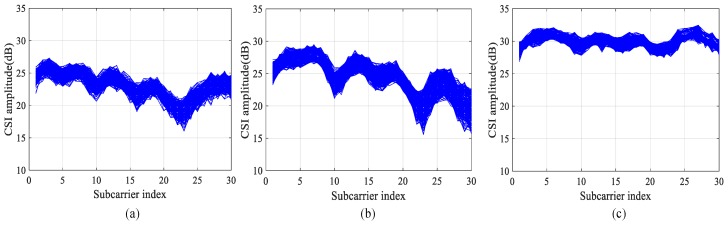
CSI Data Comparison Under Different Locations: (**a**) Unmanned Environment; (**b**) Position A; (**c**) Position B.

In order to select data better, this paper carries out a preliminary experiment. In a relatively open environment, a comparative experiment was conducted. The experimental equipment was selected to work at 5 GHz, which was more sensitive to environmental changes. As shown in Figure 4, the lower left corner is the data transmitter, the upper right corner is the data receiver, and the yellow part is the test area. First, CSI data was collected when no one was in the test area. Then, testers were asked to collect corresponding CSI data at two different locations, A and B, and collect 280 data packets at each location twice under the same circumstances. We selected the data collected from one of the antennas as the comparison antenna data, as shown in Figure 5. The horizontal coordinate in the figure is the number of subcarriers, and the vertical coordinate is the amplitude. CSI data at different positions are quite different, so different CSI data can be used to distinguish different positions.

We also chose to conduct the test in the pre-experimental environment. The wireless access point sent 280 continuous packets to the PC and collected CSI data from the reference point to obtain the initial data set. In the experiment, the tester should be stable before data collection. During the data collection process, the surrounding environment basically remains unchanged, reducing the environmental interference to some extent.

**Figure 6:**
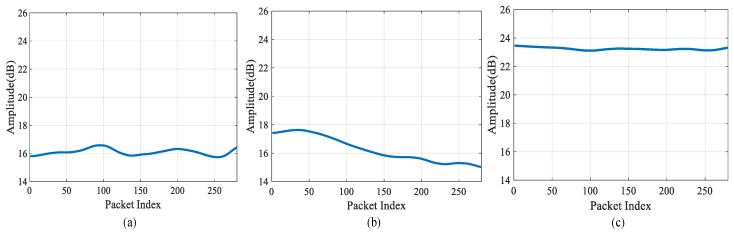
The Amplitude of Wavelet Domain Denoising: (**a**) Unmanned Environment; (**b**) Position A; (**c**) Position B.

After the initial data set is collected, the amplitude information in the CSI data is extracted, and the signal is subject to the wavelet decomposition to obtain each precise component and approximate component. After that, the precise component is subject to the threshold processed, and the processed components are used for wavelet reconstruction, and finally the denoised signal is obtained. In terms of the selection criteria of the threshold, we choose the extreme threshold principle, which means that the threshold is selected by using the minimax principle and an extremum of the minimum mean square error is produced. After that, the Daubechies (db 6) is used to decompose the signal into five layers to obtain the final waveform. As shown in Figure 6, images of three different positions in the test environment were compared after wavelet denoising.

The collected CSI amplitude information becomes smoother and more intuitive after wavelet domain denoising, and can clearly reflect the characteristics of different positions. More accurate fingerprint information can improve the positioning accuracy to some extent.

#### 3.2.2. Phase Sanitization

The CSI data collected by the experiment contain both amplitude information and phase information, but the original phase information cannot well depict the corresponding position information, which needs further processing [43]. The measured phase ${\hat{\phi }}_{i}$ for the ith subcarrier can be expressed as:

(5) $${\hat{\phi }}_{i}=\phi_{i}-2\pi \frac{k_{i}}{N}\Delta t+\beta +Z,$$

where $\phi_{i}$ is the true phase, Δt is the timing offset at the receiver, β is the phase offset caused by the carrier frequency offset, Z is some measurement noise, and $k_{i}$ is the subcarrier index of the ith subcarrier. In Intel 5300 platform, i∈(1,30) and N is the fast Fourier transform (FFT) size. Due to the above factors, Δt, β, Z ordinary Wi-Fi NICs are unable to obtain the true phase.

The original phase information cannot describe the corresponding position information well, including some noise effects caused by the environment. In this paper, the original phase information is corrected by unwinding and then linearly changed to get the phase that can better reflect the position information of the reference point.

The main idea is to eliminate Δt and β by considering phase across the whole frequency band.

First, we define two formulas a and b as follows:

(6) $$a=\frac{\phi_{n}-\phi_{1}}{k_{n}-k_{1}}-\frac{2\pi }{N}△t,$$

(7) $$b=\frac{1}{n}\sum_{j=1}^{n}\phi_{j}-\frac{2\pi △t}{nN}\sum_{j=1}^{n}k_{j}+\beta .$$

According to the IEEE 802.11n specification, the subcarrier frequency is symmetric, which indicates $\sum_{j=1}^{n}k_{j}=0$. b can be expressed as $b=\frac{1}{n}\sum_{j=1}^{n}\phi_{j}+\beta$. Subtracting the linear term $ak_{i}+b$ from the raw phase ${\hat{\phi }}_{i}$, part of the random phase shifts can be removed. We obtain a linear combination of true phases, denoted by ${\hat{\phi }}_{i}$:

(8) $${\tilde{\phi }}_{i}={\hat{\phi }}_{i}-ak_{i}-b=\phi_{i}-\frac{\phi_{n}-\phi_{1}}{k_{n}-k_{1}}k_{i}-\frac{1}{n}\sum_{j=1}^{n}\phi_{j}.$$

**Figure 7:**
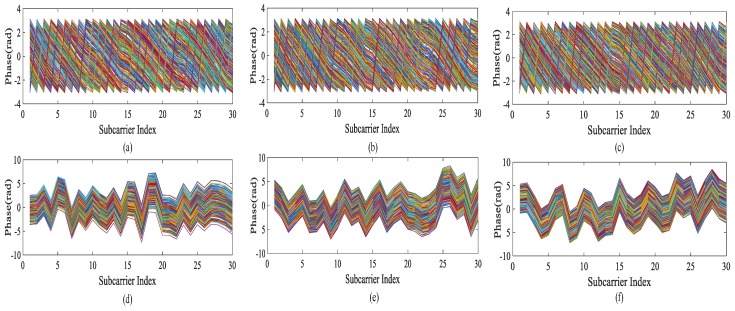
(**a**–**c**) Original Phase; (**d**–**e**) The Phase after Linear Transformation.

Figure 7a–c respectively represents the original phase images of no tester, tester at position A, and tester at position B in the pre-experimental environment. Figure 7d–f respectively represents the phase images of the above linear transformation in the pre-experimental environment with no tester, the tester at position A, and the tester at position B. From the figure, we can see that the original phase does not reflect the change of the phase caused by the change of the tester’s position, and such information cannot be stored in the fingerprint database. Through linear processing, the phase instability is effectively reduced, and the phase is converted into data that can be analyzed. In the selection of data, we choose one of the three links, which is the same as the link selected by amplitude information, to ensure the consistency of data. Using the phase information of linear transformation as the characteristic data improves the positioning accuracy to some extent.

### 3.3. Fingerprint Build

After the data preprocessing stage, we process the amplitude and phase information to correspond to the reference point one by one. The fingerprint information of each reference point includes the amplitude and phase after processing. Assuming that K reference points are selected in the environment, and the location information of each reference point is certain, CSI data of this position need to be collected at each reference point. The amplitude information is expressed as |Am|, the phase information is expressed as ϕ, and the fingerprint data of K is $K={\left|Am\right|}_{K}+\phi_{i}$, where $\phi_{i}=(\phi_{1},\phi_{2},\cdots ,\phi_{30})$. After the CSI data of each reference point are collected and processed, the fingerprint information of reference point K is mapped to the reference point, and each reference point has unique corresponding fingerprint data. The fingerprint database is denoted as F, where $F_{K}={\left|Am\right|}_{K}+\phi_{K}$, and where K=1,2,⋯,K. Finally, all fingerprint data are stored in the fingerprint database. At present, the fingerprint localization has a universal limitation: The fingerprint database is unique.

## 4. Performance Evaluation

### 4.1. Experiment Setup

**Figure 8:**
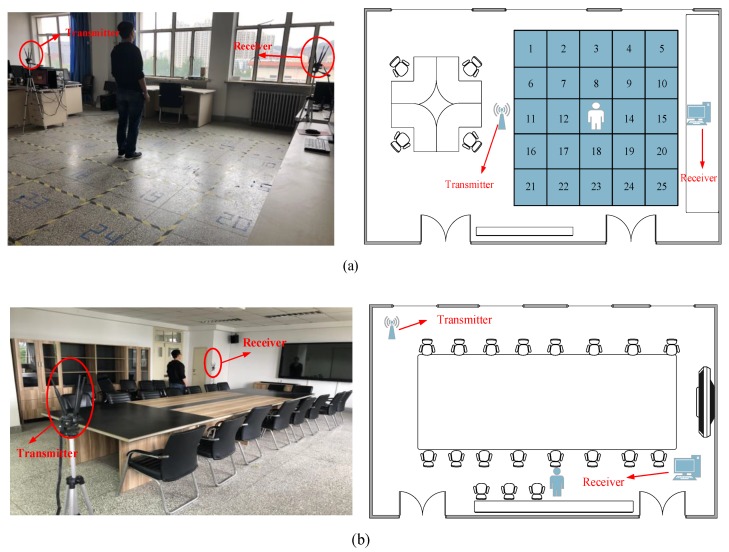
Experimental Scenarios: (**a**) Laboratory; (**b**) Conference Room.

The schematic diagram of the experimental environment is shown in Figure 8a and the whole indoor environment is 10.01 m × 6.90 m. The laboratory environment is relatively empty and the multipath effect is small. In this paper, 25 grid areas are set up to divide the experiment area into 5 × 5, a total of 25 squares. Each square area is 0.8 m. On the left of the experimental area, there is a workbench, and there is a desktop computer on the workbench with an Intel 5300 network card, which is a transmitter, a CPU of Intel Core i7-8700 (Intel, Santa Clara, CA, USA), and an operating system of Ubuntu 14.04 LTS (Canonical, London, UK). The receiver is placed on the workbench on the right side of the experimental area with the same configuration as the transmitter. The two working tables are the same height. During the test, the device is set to Monitor mode and the working frequency is 5 GHz.

We have two experimental scenarios. The first is a relatively empty laboratory, which has been used in many previous studies. As is shown in Figure 8b, the whole indoor environment is 12.01 m × 6.90 m. The conference room has many obstacles and a strong multipath effect.

### 4.2. Experimental Analysis

#### 4.2.1. Impact of the Number of Packets

In a Wi-Fi-based indoor positioning algorithm, the number of samples is one of the key factors affecting the positioning accuracy. However, the larger the sample size is, the higher the complexity of the experiment will be, and the larger the amount of data to be collected and processed. Moreover, when the number of samples reaches a certain number, the effect on the positioning accuracy is very little. Therefore, choosing an appropriate number of samples is the key to ensuring the positioning accuracy and reducing the data size.

In order to analyze the influence of sample size on the experimental mean positioning error, we conducted comparative experiments in two different environments. The tester (1.80 m in height) was selected to collect 1000, 500, 250, and 100 packets when the packet rate was fixed at 50 packets per s. Similarly, 1000 packets, 500 packets, 250 packets, 100 packets, and 50 packets were collected at a fixed rate of 10 packets per s.

**Figure 9:**
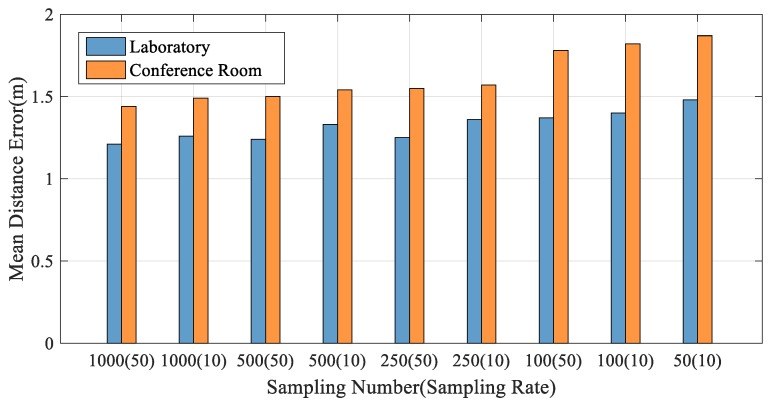
Impacts of the Number of Packets on Positioning Accuracy.

As shown in Figure 9, in the same environment, the same number of data packets are collected, and the average positioning error of the data packet with the faster packet rate is small. In the same environment and at the same packet rate, we can see that the average positioning error of 1000 packets, 500 packets, and 250 packets is not very different, but when the sample size is reduced to 100 packets and 50 packets, the average positioning error is significantly improved. This suggests that a small sample size is not sufficient to provide sufficient characteristics to reflect the overall environment. At the same time, it can be seen that the overall positioning effect in the meeting room is not as good as that in the laboratory because the experimental environment in the laboratory is relatively empty, there is more furniture in the meeting room, and the multipath effect is relatively obvious. In order to ensure the positioning accuracy and reduce the data size, 250 packets of data were collected at each reference point as sample data in this paper.

#### 4.2.2. Impact of the Number of Reference Points

In the fingerprint location algorithm, the number of reference points in the offline phase is also one of the important factors affecting the location performance. The more the number of reference points, the better the positioning will be. However, in a limited space, the more reference points there are, the more complicated data collection will be in the offline stage. The same as the number of data packets collected, when the number of reference points reach a certain number, the impact on positioning accuracy is small, but the data size becomes large, and the speed of data processing is very slow. Therefore, choosing the appropriate number of reference points is the guarantee of higher positioning accuracy and faster data processing speed.

In order to analyse the influence of the number of reference points on the positioning accuracy, we conducted a comparative experiment. In the laboratory, we selected 16, 25, 36, and 49 reference points, respectively. The reference points were divided into 4 × 4, 5 × 5, 6 × 6, 7 × 7 points with the same number of horizontal and vertical points, according to the size of the experimental environment. In this way, it can better reflect the influence of the number of reference points on the overall positioning performance. In the experiment, we selected the same testers and collected 250 packets of data at each reference point.

**Figure 10:**
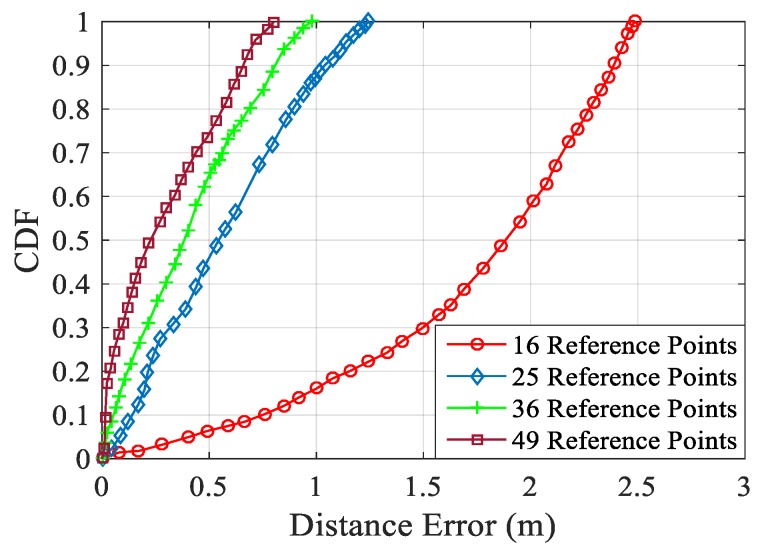
Cumulative Distribution Function (CDF) of the Number of Reference Points.

As shown in Figure 10, the more reference points there are, the smaller the positioning error will be. After selecting 16 reference points, there is a 50% probability of positioning error between 1.7 m and 2.5 m. After that, 25, 36, and 49 reference points were selected. The positioning error gradually decreased, and the positioning error constantly approached. However, the speed of positioning was also affected due to the gradual increase in the data size collected. In order to more comprehensively analyze the influence of the number of reference points on the positioning accuracy and ensure that the positioning accuracy was not affecting the positioning speed, we also compare the positioning time with the number of reference points. As shown in Table 1, with the increase of reference points, the average positioning time is increasing, the number of reference points increases from 16 to 25, and the average positioning time increases by 0.19 s, but when the number of reference points increases from 25 to 36, the average positioning time increases by 0.42 s. When the number of reference points increases from 36 to 49, the average positioning time increases by 0.34 s. The increase of average positioning time reduces the efficiency of positioning. Although the fastest positioning time exists in the whole positioning process, the impact of the overall efficiency reduction of the system is far greater than the improvement of positioning accuracy. Therefore, in this paper, 25 reference points are selected in the laboratory environment to ensure the positioning speed and the positioning accuracy to a certain extent.

For each reference point, the collected data needs to be processed separately for its amplitude and phase, and then the fingerprint library is created. Each reference point collects 250 packets of data. The number of reference points is divided into 4 × 4, 5 × 5, 6 × 6, 7 × 7 horizontal and vertical numbers, according to the actual experimental environment. The more reference points in the offline phase collection, the larger the amount of data, the higher the complexity, and the larger the fingerprint database built, which ultimately leads to a lower matching speed in the online phase. The increase of the average positioning time will reduce the efficiency of positioning. Although the entire positioning process has the fastest positioning time, it does not represent the overall processing speed of the system. The impact of the overall system processing efficiency reduction is much greater than its improvement in positioning accuracy. Therefore, this paper chooses 25 number of reference points in the laboratory environment, and the average positioning time is about 0.9 s, which not only ensures the positioning speed, but also ensures the positioning accuracy to a certain extent.

#### 4.2.3. Impact of Data Quality

In Section 2.2, the selection of collected data is introduced, and a preliminary experiment is also carried out. The selection of data has a great impact on the positioning performance. There are many ways to select amplitude information as indoor positioning, and the processing methods of amplitude are also different. In this paper, the wavelet domain denoising is selected to process amplitude information, which is of great help to improve the positioning accuracy. At the same time, in most indoor positioning algorithms, phase is rarely used, and the original phase can hardly provide corresponding features for positioning. In this paper, the fingerprint database is constructed by combining the linear transformation with the processed amplitude information and corresponding to the reference point one by one. To some extent, the noise caused by environmental factors is removed, while retaining the characteristics of each reference point.

To analyze the impact of data quality on the positioning performance, this paper set up four groups of controlled trials. The original amplitude was used as fingerprint data for positioning, the amplitude after the wavelet domain denoising was used as fingerprint data for positioning, the phase after linear transformation was used as fingerprint data for positioning, and the amplitude after the wavelet domain denoising and phase after the linear transformation in this paper were used as fingerprint data for positioning.

**Figure 11:**
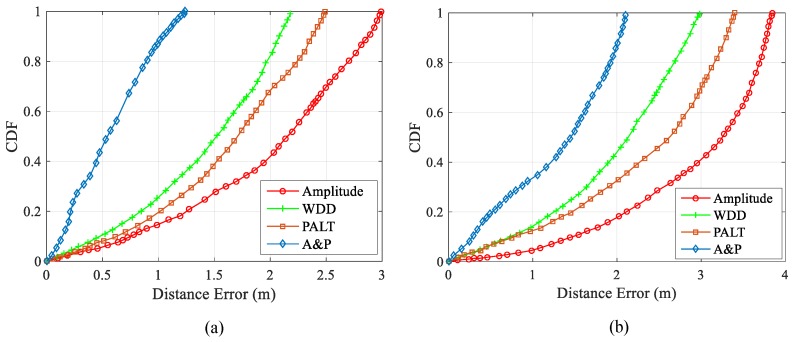
CDF of Data Quality (**a**) Laboratory; (**b**) Conference Room.

As shown in Figure 11, Amplitude refers to how the original amplitude is directly used as fingerprint data, Wavelet Domain Denoising (WDD) refers to the amplitude after the wavelet domain denoising is used as fingerprint data, Phase after Linear Transformation (PALT) refers to the phase after linear transformation is used as fingerprint data, and Amplitude and Phase (A and P) refers to how the amplitude after the wavelet domain denoising and phase after linear transformation are taken as fingerprint data in this paper. In the laboratory environment, positioning error using Amplitude is larger, followed by positioning error using PALT, and the positioning error using WDD is better than either. Nevertheless, the positioning effect is not ideal. Similarly, in the meeting room, similar to the results in the laboratory environment, the positioning effect of the three methods using amplitude or phase alone is not very ideal, while the method proposed in this paper has great advantages in both experimental environments.

### 4.3. Overrall Performance Evaluation

In order to compare the performance of different positioning algorithms, we compare the proposed algorithm with FIFS, PhaseFi, and BiLoc. In the comparative experiment, we are still conducting comparative experiments in two experimental environments, the laboratory and the meeting room. During data collection, 250 packets of data (50 packets per s) are collected at each reference point.

**Figure 12:**
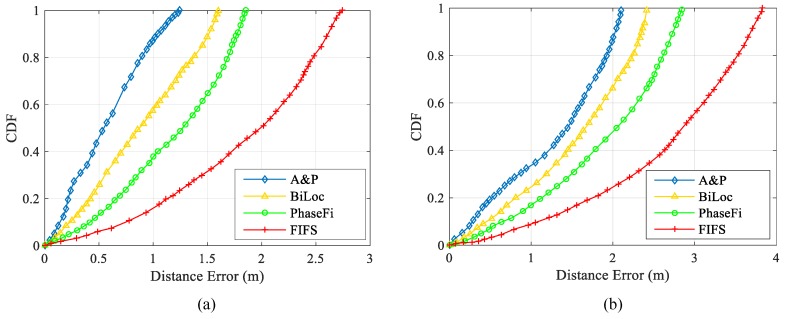
CDF of Different Localization Methods: (**a**) Laboratory; (**b**) Conference Room.

As shown in Figure 12a, in the laboratory environment, about 87% of the test position errors using the proposed method are controlled within 1 m, while 58% of the test position errors using BiLoc are controlled within 1 m, 37% of the test position errors using PhaseFi are controlled within 1 m, and 18% of the test position errors using FIFS are controlled within 1 m. Biloc’s overall performance is slightly less than the proposed methods, due to the small sample size, the amount of data the training can greatly reduce, and the average amplitude not being able to better reflect the overall location information. However, Biloc, by measuring an angle of arriving (AOA), can, to a certain extent, compensate for the effects of a small amount of sample data. PhaseFi and FIFS have poor overall positioning performance. The PhaseFi uses the deep learning method to train phase data with linear changes in the offline phase, due to the small number of samples. However, due to the small sample size, the model constructed cannot well reflect the complete location characteristics, so the positioning effect is not very ideal. Since FIFS does not carry out further processing on the collected data, the positioning error is relatively large. According to the comprehensive positioning error, BiLoc has a better positioning effect than FIFS and PhaseFi. In the offline phase, training by taking the average amplitude of data and combining it with AOA is better than training phase data with linear changes and using original data directly. Because the method proposed in this paper combines amplitude and phase information, the characteristics of samples are better than the other two methods. After data processing, some noises caused by environmental changes are removed, so that the location information is more complete and more accurate, and the positioning accuracy is greatly improved.

As shown in Figure 12b, the contrast between the meeting room environment and the laboratory environment is formed. Due to the large amount of furniture in the meeting room environment and the large influence of the multipath effect, the positioning accuracy will be reduced. However, as the method proposed in this paper is robust, certain positioning accuracy can still be maintained under the strong multipath effect, while BiLoc can control about 42% of the test position error within 1.5 m in the environment with a strong multipath effect by combining the average amplitude with AOA. About 53% of the test position errors of the proposed method are controlled within 1.5 m. Under the same conditions, the test position errors using PhaseFi and FIFS are only 30% and 16%, respectively. The experimental results show that the processed phase feature and amplitude feature can make up for the deficiency of traditional single information for positioning, and the constructed fingerprint database reflects the location information more completely and accurately, so as to achieve a better positioning effect.

In order to further evaluate the performance of the positioning algorithm, the execution time of the whole positioning system is introduced and compared in this paper. We divided the positioning into three parts: Data collection, data processing, and location estimation, and compared the method proposed in this paper with the other three methods. The data acquisition phase means the online data collection time, and the time required by the tester to conduct data collection in the position to be tested. The data processing stage means the time required to process the collected data. The location estimation stage means the time required to obtain the final positioning result by matching the location information with the fingerprint database in the online stage.

**Figure 13:**
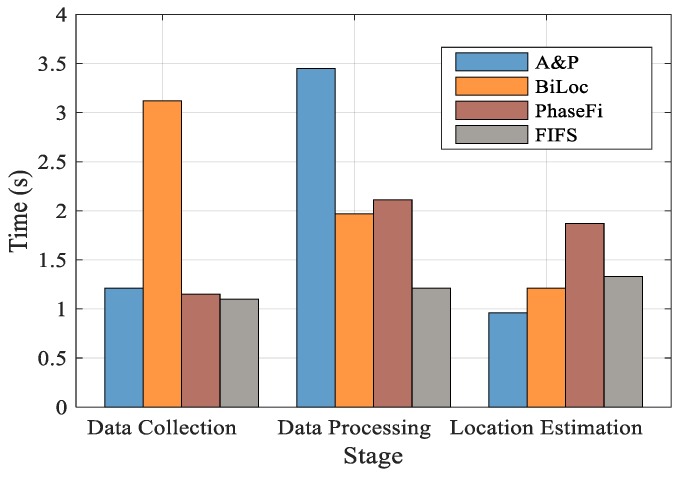
Execution Time of Different Stage.

As shown in Figure 13, in the data acquisition phase, BiLoc needs to collect CSI data and measure AOA information at the same time, so it takes a lot of time. The other three methods need similar time. Compared with the other three methods, the algorithm proposed in this paper takes more time in data processing. Because the proposed algorithm requires simultaneous processing of amplitude and phase, respectively, PhaseFi requires a large amount of time for phase processing, BiLoc requires a moment to calculate the average amplitude, while FIFS requires no special processing of data, so it consumes the least time. In the final position estimation stage, the time of position estimation is greatly reduced compared with the other three algorithms because the fingerprint database constructed by the algorithm proposed in this paper is relatively accurate. On the whole, although the method proposed in this paper consumes a lot of time in the data processing stage, it takes the least time and has the highest positioning accuracy compared with the other two methods in the position estimation stage.

## 5. Conclusions

This paper proposes a passive indoor positioning system based on CSI. In order to eliminate the interference of indoor environment on signals and obtain more accurate fingerprint data, we respectively processed CSI data collected in the offline phase. For amplitude information, the wavelet domain denoising was adopted, and for phase information, linear transformation was carried out. Finally, the fingerprint database was constructed by corresponding to the reference points one by one, which improves the positioning accuracy to some extent. We verified the system performance in two different experimental environments, the laboratory and the meeting room. Compared with other indoor positioning methods based on CSI, the method proposed in this paper has higher positioning accuracy and better accuracy.

At present, the collection and processing of data is very time consuming. The larger the data size, the higher the positioning accuracy will be. However, the larger the data size, the more difficult the data processing will be. With the application of deep learning methods, we will focus on improving the positioning accuracy and reducing the data size in future work.

## Figures and Tables

**Table 1:** Impacts of the Number of Reference Points on Positioning Time.

Number	16 Reference Points	25 Reference Points	36 Reference Points	49 Reference Points
Mean Time (s)	0.77	0.96	1.38	1.72
The Fastest Time (s)	0.63	0.81	1.22	1.52
